# No effect of 6-month intake of glucosamine sulfate on Modic changes or high intensity zones in the lumbar spine: sub-group analysis of a randomized controlled trial

**DOI:** 10.1186/1477-5751-11-13

**Published:** 2012-08-17

**Authors:** Philip Wilkens, Kjersti Storheim, Inger Scheel, Linda Berg, Ansgar Espeland

**Affiliations:** 1 Department of Orthopaedics, Oslo University Hospital, FOU, OS, BD, Bygg 73, Kirkeveien 166, 0460 Oslo, Norway; 2 Department of Orthopaedics, University of Oslo, Kirkeveien 166, 0450 Oslo, Norway; 3 Norwegian Research Centre for Active Rehabilitation (NAR), Hjelp24 NIMI, Pb. 3843 Ullevål Stadion, Sognsveien 75 D, 0805 Oslo, Norway; 4 Communication- and Research Unit for Musculoskeletal Disorders, FORMI, Oslo University Hospital, Ullevål, Building 37b, 0407 Oslo, Norway; 5 Norwegian Knowledge Centre for the Health Services, PO Box 7004 St. Olav’s plass, 0130 Oslo, Norway; 6 Department of Radiology, Haukeland University Hospital, Jonas Lies vei 65, 5021 Bergen, Norway; 7 Section for Radiology, Department of Surgical Sciences, University of Bergen, Jonas Lies vei 65, 5021, Bergen, Norway

**Keywords:** Glucosamine sulfate, High intensity zone, Low back pain, Lumbar spine, Magnetic resonance imaging, Modic changes, Randomized trial, Treatment effect

## Abstract

**Background:**

The underlying pathology and natural course of Modic changes (MC) in the vertebral body marrow and high intensity zones (HIZs) in the annulus fibrosus is not completely clarified. These findings on magnetic resonance imaging (MRI) have initiated different treatments with little or unclear effect. In a randomized trial (n = 250), glucosamine sulfate (GS) had no effect on low back pain related disability. GS could still have an effect on MC and HIZ. In this sub-study, 45 patients from the trial who had MC and/or HIZ at pre-treatment underwent follow-up MRI. The aim was to examine the course of MC and HIZ and to compare this course between groups treated with 6-month intake of oral GS versus placebo.

**Results:**

Of 141 pre-treatment MC in 42 (of 45) patients, 29 (20.6%) MC in 18 patients had altered type and 14 MC in 9 patients had altered size (decreased for 1 MC) 6-18 months later: odds ratio (OR) for type vs. size alterations 4.0; 95% confidence interval (CI) 1.2-17.7. No MC resolved. HIZ vanished from 3 of 23 discs in 3 of 21 patients with pre-treatment HIZ. Ten new MC (all type I or I/II) occurred in 8 patients and 2 new HIZs in 2 patients. The GS group (n = 19) and placebo group (n = 26) did not differ in proportions of MC with decreased (OR 1.6; 95% CI 0.4-6.1) or increased type I dominance at follow-up (OR placebo:GS 2.4; 95% CI 0.6-9.7), or with increased size (OR 1.0; 95% CI 0.2-4.7). HIZ vanished from 1 of 8 discs in 1 of 8 patients in the GS group vs. 2 of 15 discs in 2 of 13 patients in the placebo group (OR 0.8; 95% CI 0.02-12.2).

**Conclusions:**

In this sub-group analysis of a placebo-controlled trial, the effect of GS on MC and HIZs was no different from the effect of the placebo intervention. MC and HIZs remained mostly unchanged during the 6-18 months study period. Some short term changes did occur and MC more often altered type than size.

**Trial registration:**

NCT00404079 at http://www.clinicaltrial.gov.

## Background

Vertebral body marrow changes (Modic changes (MC)) in the lumbar vertebrae and high intensity zones (HIZs) in the lumbar discs are frequent findings on magnetic resonance imaging (MRI) [1]. Relationship has been suggested between these findings and low back pain (LBP) [2-5]. MC can be classified into three types (I to III) [6,7]. Histological examination of MC type I demonstrates disrupted and fissured endplates with regions of degeneration, regeneration, reactive bone formation, endplate edema and vascular granulation [7,8]. MC type II displays endplate disruption and fatty degeneration on histological examination and MC type III shows sclerosis [7]. MC contain various enzymes, inflammatory mediators (e.g. tumor necrosis factor (TNF)) and nociceptive nerve fibers [9-11]. Their origin is unknown, but mechanical stress, low grade infection secondary to disc herniation or some auto-immune reaction are proposed mechanisms [12]. MC are suggested to follow a sequential pathway of a common pathological process starting with type I followed by type II. Type I is often considered an unstable lesion that tends to alter over time, while type II is considered more stable [7,13-18]. Nonetheless, type II may change back to normal, return to type I or develop into type III [17,19]. The stability of type III remains uncertain [14]. Mixed types I/II and II/III have also been identified [20,21].

An HIZ is a focal area of high signal intensity within the posterior part of the annulus of a disc [22]. This finding often occurs in the early stages of disc degeneration and may be related to a faster subsequent degeneration [23]. It is hypothesized to represent a collection of mucoid fluid within fissures of the annulus or a reflection of the edge neovascularization of posterior annulus or a healing annulus tear. These annular tears are separations between annular fibers, separations of annular fibers from their vertebral insertions, or breaks through these fibers in any orientation, involving one or more layers of the annular lamellae [24]. The annulus fibrosus is innervated by the recurrent meningeal nerve and by the small branches from the ventral ramus of the somatic spinal nerve [25]. HIZs may affect these nerve endings by acid metabolites contained in the disk material and could therefore produce LBP or referred pain even in the absence of actual nerve root compression [8].

The underlying pathology and natural history of MC and HIZs is not completely clarified [12]. The identification of these findings has led to different treatments (e.g. antibiotic, intradiscal injection, surgery) with limited evidence of effect [26,27]. In a randomized controlled trial (RCT) of 250 chronic LBP patients, we found no effect of glucosamine sulfate (GS) on LBP-related disability [28]. GS may still have an effect on structural changes that was not detected by disability evaluation within the 1-year follow-up period. GS may target IL-1β [29], a cytokine associated with inflammation in the osteoarthritic degenerative process [30]. Because MC and HIZ are plausible markers of an osteoarthritic degenerative process and are related to secretion of proinflammatory mediators [31], they may contain IL-1β.

In this study, a sub-group of patients (n = 45) with MC and/or HIZ at pre-treatment in our RCT underwent MRI 6-18 months later [28]. The purpose was to examine the course of MC and HIZ and to compare this course between groups treated with 6-month intake of oral GS versus placebo. We hypothesized that GS a) facilitates MC type conversion from the more inflammatory type I to types II or III (or to normal) and prevents switch of other MC types back to type I, b) reduces MC size or prevents increased size, and c) causes HIZs to disappear.

## Results

All 45 included patients (mean age 45 years, range 31-65, 20 females 44.4%) had complete MRI data at all studied endplates (n = 450) and discs (n = 225). MRI was obtained 0-12 (median 1) months prior to the start of the treatment period (< 50 days prior to the start of that period in 35 of 45 patients, 77.8%). Follow-up MRI was performed 0-12 (median 2) months after the end of the 6-month treatment period, i.e. 6-18 (median 8) months after the pre-treatment MRI.

### Pre-treatment findings in total sub-sample

Table 1 presents the frequency and type of MC pre- and post-treatment for the 45 patients in the total sub-sample. Pre-treatment MRI showed MC at 141 (31.3%) of 450 endplates in 42 patients, most often type II (76/141, 53.9%) or type I (26/141, 18.4%; Table 1). Tables 1, 2, 3, 4 show the number/type, number of affected levels, location and size of pre-treatment MC. MC size concerned anteroposterior (AP) diameter and craniocaudal (CC) extension of the MC as a proportion of the AP endplate diameter and vertebral body height, respectively.

**Table 1:** Frequency of Modic changes (MC) in total sub-sample and by treatment group (glucosamine or placebo)

**Total sub-sample (45 patients, 450 lumbar endplates)**											
		**Pre-treatment**									
**Post-treatment**		No MC	MC I	MC I/II	MC I/III	MC II	MC II/I	MC II/III	MC III	MC III/I	MC III/II
	Total	**309**	**26**	**17**	**2**	**76**	**11**	**4**	**2**	**1**	**2**
No MC	299	**299**	0	0	0	0	0	0	0	0	0
MC I	25	**7**	**16**	0	0	**1**	0	0	0	**1**	0
MC I/II	27	**3**	**7**	**13**	0	**2**	**2**	0	0	0	0
MC I/III	1	0	0	0	**1**	0	0	0	0	0	0
MC II	71	0	0	**1**	0	**69**	**1**	0	0	0	0
MC II/I	20	0	**3**	**3**	**1**	**4**	**8**	**1**	0	0	0
MC II/III	3	0	0	0	0	0	0	**3**	0	0	0
MC III	0	0	0	0	0	0	0	0	0	0	0
MC III/I	0	0	0	0	0	0	0	0	0	0	0
MC III/II	4	0	0	0	0	0	0	0	**2**	0	**2**
**Glucosamine group** (19 patients, 190 lumbar endplates)											
		**Pre-treatment**									
**Post-treatment**		No MC	MC I	MC I/II	MC I/III	MC II	MC II/I	MC II/III	MC III	MC III/I	MC III/II
	Total	**126**	**18**	**8**	**2**	**28**	**5**	**1**	**2**	**0**	**0**
No MC	121	**121**	0	0	0	0	0	0	0	0	0
MC I	15	**3**	**12**	0	0	0	0	0	0	0	0
MC I/II	15	**2**	**4**	**7**	0	**1**	**1**	0	0	0	0
MC I/III	1	0	0	0	**1**	0	0	0	0	0	0
MC II	28	0	0	**1**	0	**26**	**1**	0	0	0	0
MC II/I	7	0	**2**	0	**1**	**1**	**3**	0	0	0	0
MC II/III	1	0	0	0	0	0	0	**1**	0	0	0
MC III	0	0	0	0	0	0	0	0	0	0	0
MC III/I	0	0	0	0	0	0	0	0	0	0	0
MC III/II	2	0	0	0	0	0	0	0	**2**	0	0
**Placebo group** (26 patients, 260 lumbar endplates)											
		**Pre-treatment**									
**Post-treatment**		No MC	MC I	MC I/II	MC I/III	MC II	MC II/I	MC II/III	MC III	MC III/I	MC III/II
	Total	**183**	**8**	**9**	**0**	**48**	**6**	**3**	**0**	**1**	**2**
No MC	178	**178**	0	0	0	0	0	0	0	0	0
MC I	10	**4**	**4**	0	0	**1**	0	0	0	**1**	0
MC I/II	12	**1**	**3**	**6**	0	**1**	**1**	0	0	0	0
MC I/III	0	0	0	0	0	0	0	0	0	0	0
MC II	43	0	0	0	0	**43**	0	0	0	0	0
MC II/I	13	0	**1**	**3**	0	**3**	**5**	**1**	0	0	0
MC II/III	2	0	0	0	0	0	0	**2**	0	0	0
MC III	0	0	0	0	0	0	0	0	0	0	0
MC III/I	0	0	0	0	0	0	0	0	0	0	0
MC III/II	2	0	0	0	0	0	0	0	0	0	**2**

**Table 2:** Frequency of MC by number of affected endplates per patient (45 patients, 450 lumbar endplates)

**Number of endplates affected by MC**	**Pre-treatment frequency of MC (any type)**	**Post-treatment frequency of MC (any type)**
1	7	6
2	12	10
3	4	6
4	9	8
5	4	4
6	1	3
7	4	3
8	1	2
9	0	0
10	0	0
Total	141	151

MC, Modic changes.

**Table 3:** Frequency of MC by lumbar level (45 patients, 225 levels, 2 endplates per level)

	**Pre-treatment**		**Post-treatment**	
**Level**	**Number of patients with MC**	**Number of endplates affected by MC**	**Number of patients with MC**	**Number of endplates affected by MC**
L1-L2	5	6	5	6
L2-L3	10	16	11	17
L3-L4	14	23	17	27
L4-L5	24	43	26	45
L5-S1	31	53	34	56
Total	Not applicable	141	Not applicable	151

MC, Modic changes.

**Table 4:** Pre-treatment frequency of Modic changes by size (45 patients, 450 lumbar endplates)

**Modic changes type I** (26 endplates)				
	**AP diameter**			
**CC extension**	<1/4	1/4 -1/2	>1/2	Total
<1/10 (minimal / small dots)	1	1	1	3
<1/4	1	4	4	9
1/4 - 1/2	0	1	7	8
>1/2	0	0	6	6
**Modic changes type I/II** (17 endplates)				
	**AP diameter**			
**CC extension**	<1/4	1/4 -1/2	>1/2	Total
<1/10 (minimal / small dots)	0	1	1	2
<1/4	0	1	4	5
1/4 - 1/2	0	1	4	5
>1/2	0	0	5	5
**Modic changes type II** (76 endplates)				
	**AP diameter**			
**CC extension**	<1/4	1/4 -1/2	>1/2	Total
<1/10 (minimal / small dots)	12	3	1	16
<1/4	11	15	15	41
1/4 - 1/2	0	3	11	14
>1/2	0	0	5	5
**Modic changes type II/I** (11 endplates)				
	**AP diameter**			
**CC extension**	<1/4	1/4 -1/2	>1/2	Total
<1/10 (minimal / small dots)	0	0	0	0
<1/4	0	2	1	3
1/4 - 1/2	0	0	7	7
>1/2	0	0	1	1
**Modic changes all other types (I/III, II/III, III, III/I or III/II)** (11 endplates)				
	**AP diameter**			
**CC extension**	<1/4	1/4 -1/2	>1/2	Total
<1/10 (minimal / small dots)	0	0	0	0
<1/4	0	0	1	1
1/4 - 1/2	0	0	3	3
>1/2	0	0	7	7

AP diameter, maximal anteroposterior diameter of Modic changes as a proportion of the anteroposterior endplate diameter; CC extension, maximal craniocaudal extension of Modic changes as a proportion of the vertebral body height.

Pre-treatment MRI showed HIZ in 23 (10.2%) of 250 discs in 21 patients, at L3-L4 (5 of 23 discs), L4-L5 (9 of 23 discs), and L5-S1 (also 9 of 23 discs, 39.1%). HIZ affected one disc in 20 patients and three discs in 1 patient.

### Post-treatment changes in total sub-sample

**Figure 1:**
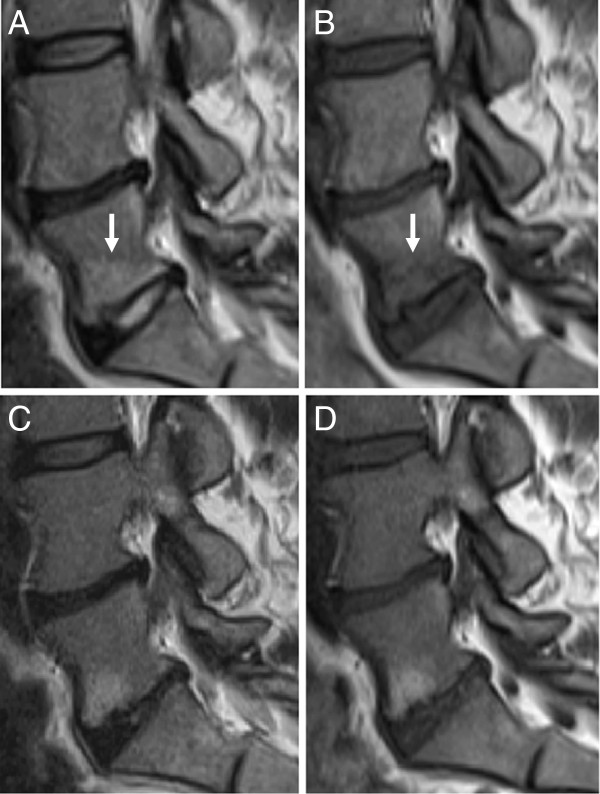
**Altered type of Modic changes.** Initial (**A-B**) and follow-up (**C-D**) sagittal magnetic resonance images of one patient. Type I Modic changes (**arrows;** high signal on T2-weigthed image **A,** low signal on T1-weighted image **B**) alters to type II (high signal on T2- and T1-weighted images **C** and **D;** images not shown revealed alteration to type II/I).

**Figure 2:**
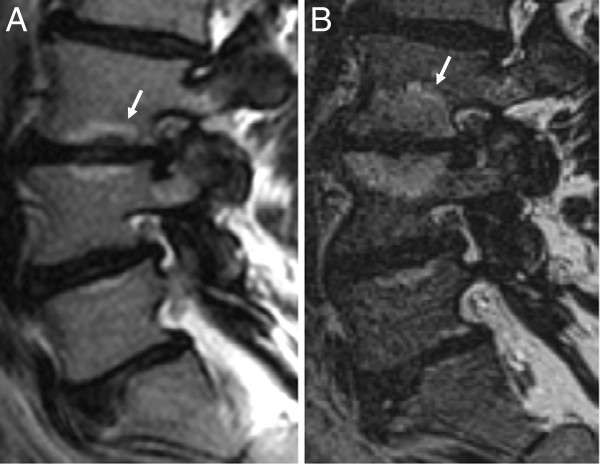
**Increased size of Modic changes.** Initial (**A**) and follow-up (**B**) sagittal T2-weigthed magnetic resonance images of one patient. Modic changes at L3/L4 increase in craniocaudal extent from <1/4 to >1/2 of the vertebral body height from image **A** to image **B** (**arrows**).

At follow-up 6-18 months after their initial MRI, 21 of all 42 patients with pre-treatment MC had altered MC type and/or MC size. Of the 141 pre-treatment MC, 29 (20.6%) MC had altered type (Figure 1), 13 MC had increased size (Figure 2), and 1 MC had reduced size. Type and size alterations of MC affected 18 vs. 9 of 42 patients, respectively: p = 0.02; odds ratio (OR) 4.0, 95% confidence interval (CI) 1.2-17.7. MC size did not change at the L1-L2 endplates.

**Figure 3:**
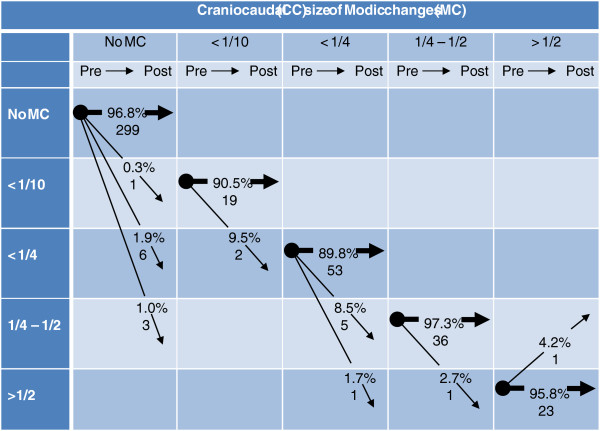
**Pre- and post-treatment craniocaudal size of Modic changes at 450 endplates in 45 patients.** Arrows indicate size development. Numbers are percentages and numbers of endplates.

Tables 1, 2, 3 show the number/type, number of affected levels, and location of MC post-treatment. The type changed for 10 of 26 MC type I and 7 of 76 MC type II (Table 1). Four MC in 4 patients increased their AP diameter. Nine MC in 9 patients had increased CC size and 1 MC had decreased CC size (Figure 3). No MC resolved completely (to no MC). New MC occurred in 8 patients at 10 (3.2%) of 309 endplates with no pre-treatment MC. These were 7 new MC type I at levels L5-S1 (3 MC), L3-L4 (3 MC) and L2-L3 (1 MC) and 3 new MC type I/II at levels L4-L5 (2 MC) and L3-L4 (1 MC). Only 1 of the 10 new MC had CC size <1/10 (Figure 3).

**Figure 4:**
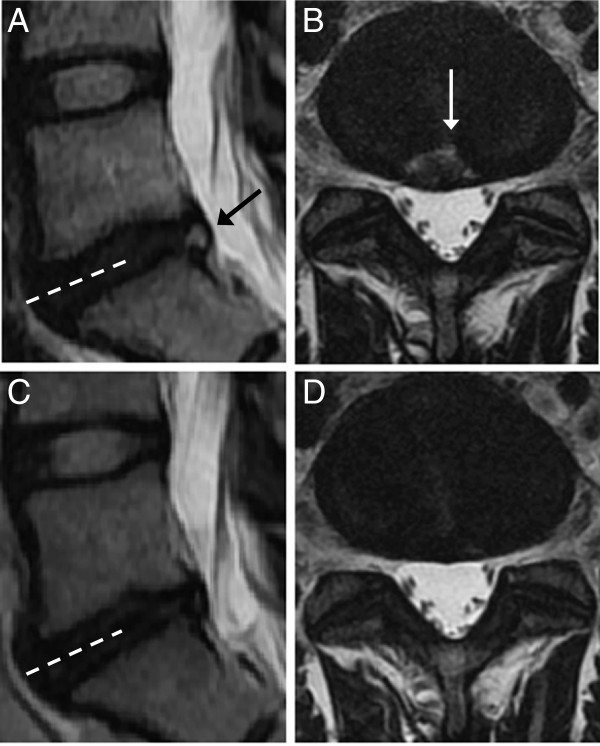
**Resolving high intensity zone.** Initial (**A-B**) and follow-up (**C-D**) T2-weighted magnetic resonance images of one patient. Image plane **B** is marked on **A** and image plane **D** is marked on **C** (**stippled lines**). High intensity zone in the L5/S1 disc on initial sagittal (**A**) and axial (**B**) images (**arrows**) is resolved on later sagittal (**C**) and axial (**D**) images.

At follow-up, HIZ had disappeared from one L5-S1 disc (Figure 4) and two L4-L5 discs in 3 patients but was still present in 20 of 23 discs in 18 of 21 patients with pre-treatment HIZ. New HIZ had developed in 2 patients, in the L5-S1 disc.

### Comparison of changes between treatment groups

**Figure 5:**
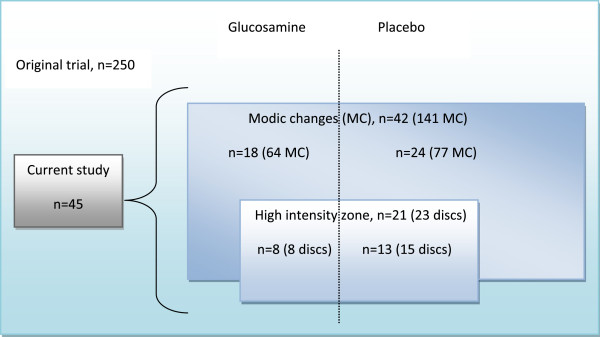
**Current sub-sample and pre-treatment findings by treatment group.** Shown are numbers of Modic changes (MC) and of discs with high intensity zone (HIZ) by treatment group (glukosamine or placebo) in the current sub-sample of 45 patients with MC and / or HIZ from a trial of 250 chronic low back pain patients; n denotes number of patients.

Pre-treatment, in the GS- (n = 19) and placebo group (n = 26), 18 and 24 patients had MC and 8 and 13 patients had HIZ, respectively (Figure 5). Pre-treatment, the GS- and placebo group were comparable in prevalence (per endplate), types, and sizes of MC and in prevalence (per disc) of HIZ (p > 0.67). The frequency of MC pre- and post-treatment differentiated by treatment groups is presented in Table 1. Post-treatment, the GS- and placebo group did not differ in proportions of MC with decreased type I dominance (defined in Methods) (OR GS:placebo 1.6, 95% CI 0.4-6.1; p = 0.46), increased type I dominance (OR placebo:GS 2.4, 95% CI 0.6-9.7; p = 0.22), or increased MC size (OR 1.0, 95% CI 0.2-4.7; p = 0.97).

At follow-up, HIZ had disappeared from 1 of 8 discs in 1 of 8 patients in the GS group vs. 2 of 15 discs in 2 of 13 patients in the placebo group (OR 0.8; 95% CI 0.02-12.2, p = 0.77). One new HIZ occurred in each group.

## Discussion

In the present study, almost 80% of the MC and more than 85% of the HIZs remained stable in the 6-18 month study period. No MC resolved, but new MC, all type I or type I/II, developed at 10 of the 450 studied endplates. MC altered type at 29 and size at 14 of 141 affected endplates. HIZs resolved in 3 of 23 affected discs and occurred in 2 new discs. GS did not alter the presence or size of the MC, nor the presence of HIZ compared to placebo.

To the authors’ knowledge, this is the first trial to test the effect of GS on MC and HIZ and therefore no directly comparable data exists. Other treatments of patients with LBP and findings of MC or HIZ have been tested for clinical effect but not for effect on MC or HIZ. An uncontrolled pilot study found clinical effect of antibiotics in patients with LBP and MC type I [26]. Another study of 120 patients with LBP and MC type I or II indicated short-term clinical effect of intradiscal steroid injection [27]. LBP patients with HIZ have been treated with other interventions like intradiscal electrothermal therapy and intradiscal radiofrequency thermocoagulation without clear conclusions [32-34].

Several reasons may explain the lack of difference in effect on MC and HIZ between GS- and placebo group. GS may be ineffective as modifier of the potential markers of inflammatory pain and secretion of proinflammatory mediators associated with MC and HIZ. Previous research has demonstrated the inability of GS to reduce LBP or LBP-related disability [28]. GS may slow down the destruction of cartilage in osteoarthritis (OA) by inhibiting the pro-inflammatory IL-1β [29]. IL-1β is associated with cartilage destruction in OA [35]. However, opposite to our assumptions, IL-1 β may not be pathologically relevant for MC or HIZ. Furthermore, 6-month glucosamine exposure may be too short time period to impact an area with limited direct blood supply. It is also possible that GS does not reach the target area because of either low concentration in the blood stream or insufficient blood supply to the lumbar vertebras and discs. However, the lack of demonstrable difference may also arise from inadequate sample size. A larger sample of MC and especially of HIZs would have made it easier to detect any smaller effect of GS on the rather slow natural course of these findings.

No MC vanished during the study period. MC are not necessarily everlasting as population research has reported resolution of MC [36]. However, the research is conflicting as others have found limited, or no evidence of resolution of established MC [17,19]. Different type of sample (general population versus LBP patients) and sample size (> 300 versus < 50 patients) may explain some of the discrepancy [17,19,36].

The development of 10 new MC (in 8 of 45 patients) confirmed that MC often surface in patients with LBP [36]. All new MC were predominantly type I, which may support the notion that MC type I is the start point for the MC evolution [12]. Several MC, more type I than type II converted into a different type, indicating that MC are viable to change in as short term as 6 months to 1 year. Previous research has shown that MC may convert over 3 to 5 years after discectomy [37]. In addition, any type of MC may be more prone to switch between types than previously thought [7].

Our data confirmed that MC type I is less stable than MC type II [13,17]. However, almost 80% of the MC did not change, which is also comparable with previous studies [20]. The MC size in terms of AP diameter and CC extension was more stable than the type of MC. It is noteworthy that also small MC had stable extension and did not tend to come and go. This was different from previous results that were not based on direct comparison of initial and follow-up images [36]. The most common places for MC to occur were at the L5-S1 and L4-L5 endplates, which is in line with previous reports [14].

The most common locations for HIZ were also L5-S1 and L4-5, which follows previous reports [38]. More than 85% of the discs with HIZ remained stable throughout the study, which is also in line with earlier research [39]. On the other hand, HIZ was in most cases (more than 95%) present at one disc space only. The occurrence of HIZ at one level only is also comparable with past research [38-42].

The present study has several strengths. It included a potentially important and distinct sub-group of LBP sufferers with MC and HIZ among the greater population of unspecific, longstanding low back pain. Two independent readers rated the MRIs using established criteria, a third independent reader resolved any disagreements, and all readers were blinded to age, gender, treatment and clinical information. Changes in MRI findings were rated by comparing initial and follow up images. This approach reflects clinical practice and is optimal for rating changes in MRI findings over time [43,44]. Assessment of follow-up images blinded to the initial images, may introduce unwanted variation in the rating of any alterations.

Study limitations require attention. This sub-study of the original RCT should be considered exploratory in nature. It had small sample size and was not based on a separate power calculation. The wide time range (more than 50 days) between some of the pre-treatment MRIs (10 patients) and the start of the treatment may have clouded potential alterations in MC and HIZ due to GS. Furthermore, all readers knew that all of the images were from patients with MC and/or HIZ, and this knowledge may have influenced the evaluation. However, an abundant number of normal as well as abnormal spinal levels were evaluated. Slight variability in MRI technique introduced heterogeneity, but reflects clinical practice. We focused on two MRI abnormalities only and did not address other potentially relevant degenerative findings like disc- or facet degeneration, discs bulges or disc herniations. The location of MC within the endplate was not assessed. HIZ was not confirmed using discography and we did not apply contrast enhanced T1-weighted MRI, which may be more sensitive to detect HIZ than T2-weighted MRI [45,46]. However, Munter et al found no increased sensitivity to detect HIZ with contrast enhancement [47]. New biochemical MRI methods exist for evaluating the intervertebral disc, such as T2 mapping, T2* mapping and diffusion weighted imaging [48-50]. These methods may be more sensitive to changes under therapy than the morphological MRI techniques used in our study.

## Conclusions

GS had no clear effect on MC or HIZ in this group of LBP patients. Regardless of intervention, most findings of MC and HIZ remained stable during the 6-18 months study period. However, short term changes in MC and HIZs did occur. Based on this study, it is likely that the MC development starts with type I, that MC more often alter type than size, and that small MC may be equally stable as larger MC.

## Methods

This study included a sub-sample of 45 patients who had any type of MC and/or HIZ on lumbar MRI at inclusion in a prospective double-blind RCT comparing GS to placebo as treatment for chronic LBP [28] (Figure 5). The original RCT included 250 patients and was registered at http://www.clinicaltrial.gov under the identifier NCT00404079. The study was carried out in compliance with the Helsinki Declaration and it was approved by The Norwegian Medicines Agency, The Regional Committees for medical research ethics in east Norway and the Data Inspectorate. Written informed consent was obtained from all participants prior to enrollment.

### Eligibility criteria and intervention

The 45 patients were recruited from 12.6.2007 to 16.7.2008. They were asked to participate in the present study by undergoing follow-up MRI and all agreed. As detailed elsewhere [28], inclusion criteria in the original trial were age > 25 years, primary complaint of nonspecific LBP, LBP for at least six months, and summed score of at least 3 out of 24 points on Roland Morris Disability Questionnaire. Exclusion criteria included worse leg pain than back pain, symptomatic disc herniation or spinal stenosis, previous lumbar fracture or surgery, pregnancy or breastfeeding, seafood allergy, ongoing psychiatric or somatic disease potentially influencing their pain and use of any type of glucosamine 1 year prior to enrollment. The intervention consisted of three capsules (500 mg each) of GS or placebo every day for six months. Initiation of new therapies was not permitted during the intervention period, but the patients were allowed to maintain established management and/or use rescue medication.

### MRI evaluation

The 45 patients underwent 1.5 T MRI at different imaging institutions. Follow-up MRI was taken from 1.2.2008 to 13.5.2009. The same scanner was used pre- and post-treatment for 21 (46.7%) patients. All 90 MRIs included sagittal T1- and T2-weighted scans of the whole lumbar spine and axial T1- or T2-weighted scans of at least the three lower lumbar levels. Sagittal T1-weighted images were turbo spin echo (TSE) images (repetition time (TR) / echo time (TE), 400-911 ms / 8-14 ms) or, for 10 pre- and 8 post-treatment MRIs, fast fluid-attenuated inversion-recovery images (TR / TE, 1989-1999 ms / 20 ms). Sagittal T2-weighted images were TSE images (TR / TE, 2500-5930 ms / 89-125 ms) or, for 11 post-treatment MRIs, spatial and chemical shift encoded excitation images (1500 ms / 251 ms).

Two observers, blinded to clinical data and treatment group, independently rated MC and HIZs for the 45 pre-treatment MRIs, presented de-identified in a random order. The observers were an experienced radiologist (AE) and a chiropractor (PW) experienced in evaluating MC and HIZ. In two pilot studies, they had interpreted and discussed 20 lumbar MRIs from another study to improve their ratings of MC and HIZ. The MC classification has demonstrated to be reliable to apply for observers of varying experience [21,51,52]. In our study, interobserver agreement was good to very good for MC (yes/no, kappa 0.69-1.00) and fair to good for HIZ (yes/no, kappa 0.35-0.73) at L3/L4, L4/L5 and L5/S1 on pre-treatment MRI (kappa not calculated at L1/L2 or L2/L3 due to low prevalence of “yes” [53]).

MC were evaluated at each of the ten endplates L1-S1. They were graded into MC type 0 (no MC), I (hypointense T1-signal and hyperintense T2-signal), II (hyperintense T1-signal and iso- or hyperintense T2-signal), III (hypointense T1-signal and hypointense T2-signal) and mixed types (e.g. I/II, listing the most extensive type first) [7,8,24,54]. Maximal anteroposterior (AP) diameter of the MC was recorded as <1/4, ¼-1/2 or >1/2 of the AP endplate diameter. Maximal craniocaudal (CC) extent of the MC was recorded as <1/10 (minimal/small dots), <1/4, ¼-1/2 or >1/2 of the vertebral body height [51]. HIZ was identified on T2-weighted images as an area of high signal intensity in the posterior annulus fibrosus that was brighter than the nucleus pulposus and was surrounded superiorly, inferiorly and anteriorly by the low-intensity (black) signal of the annulus [55]. HIZ was noted as present or not present at each of the five disc levels L1-S1. Anterior annular fissure was not assessed.

In all cases of disagreement between the two observers a third observer (a second experienced radiologist, LB) independently examined the images. The majority view (or median rating for size of MC) was taken as the conclusive rating. If all three observers disagreed on type of MC they re-evaluated the images in consensus and reported a shared conclusive result. The conclusive rating was first determined for all pre-treatment scans and was noted on a form. Then, on separate copies of this form observers 1 and 2 independently (and still blinded to clinical data and treatment group) reported changes in rating from pre- to post-treatment MRI based on direct comparison of the two sets of images. Again, in all cases of disagreement a conclusive majority or consensus rating was achieved with the observer 3.

### MC and HIZ outcomes

The outcomes used to test the effect of GS were the proportions of a) MC with decreased or increased MC type I dominance post-treatment, b) MC with decreased or increased size (at least one category change in AP diameter and/or CC extent), and c) discs with HIZ where HIZ had disappeared. The order of more to less MC type I dominance was defined by the following four combined MC type categories: I, I/II-III, II-III/I, all other types.

### Statistical analysis

The frequency of MC and HIZ at pre- and post-treatment MRI and the frequency of alterations of MC and HIZ from pre-treatment to post-treatment were calculated with frequency tables and cross-tabulations. Proportions of patients with altered type vs. altered size of MC were compared using McNemar’s test and by computing OR with mid-P exact 95% CI [56]. Proportions of changed MC were compared between treatment groups by calculating ORs with 95% CIs adjusted for intracluster correlations between different pre-treatment MC in the same patient (Rao-Scott method) [57]. Since only one patient had HIZ at more than one disc (i.e. clustered data), proportions of patients (not discs) with vanished HIZ were compared using OR with mid-P exact 95% CI. All p-values are 2-sided and the significance level was 5%. Analyses were performed with SPSS version 18.0 for Windows (SPSS, Inc., Chicago, Illinois) and WINPEPI version 11.22 ( http://www.brixtonhealth.com/pepi4windows.html).

## Abbreviations

AP: Anteroposterior; CC: Craniocaudal; CI: Confidence interval; GS: Glucosamine sulfate; HIZ: High intensity zone; LBP: Low back pain; MC: Modic changes; MRI: Magnetic resonance imaging; OA: Osteoarthritis; OR: Odds ratio; RCT: Randomized controlled trial; TE: Echo time; TNF: Tumor necrosis factor; TR: Repetition time; TSE: Turbo spin echo.

## Competing interests

The authors declare that they have no competing interests.

## Authors’ contributions

PW participated in the study design, data acquisition, and data analysis, helped to coordinate the study, evaluated the MRIs, and drafted the manuscript. KS participated in the study design, data acquisition, and data analysis, and helped to coordinate the study and draft the manuscript. IS participated in the study design and data interpretation and helped to draft the manuscript. LB evaluated the MRIs in cases of disagreement between PW and AE, and helped to interpret the data and draft the manuscript. AE participated in the study design, data acquisition, and data analysis, evaluated the MRIs, and helped to coordinate the study and draft the manuscript. All authors read and approved the final manuscript.
